# Skn-1a/Pou2f3 functions as a master regulator to generate Trpm5-expressing chemosensory cells in mice

**DOI:** 10.1371/journal.pone.0189340

**Published:** 2017-12-07

**Authors:** Junpei Yamashita, Makoto Ohmoto, Tatsuya Yamaguchi, Ichiro Matsumoto, Junji Hirota

**Affiliations:** 1 Department of Life Science and Technology, Graduate School of Life Science and Technology, Tokyo Institute of Technology, Yokohama, Japan; 2 Monell Chemical Senses Center, Philadelphia, United States of America; 3 Center for Biological Resources and Informatics, Tokyo Institute of Technology, Yokohama, Japan; Meiji Daigaku - Ikuta Campus, JAPAN

## Abstract

Transient receptor potential channel M5 (Trpm5)-expressing cells, such as sweet, umami, and bitter taste cells in the oropharyngeal epithelium, solitary chemosensory cells in the nasal respiratory epithelium, and tuft cells in the small intestine, that express taste-related genes function as chemosensory cells. Previous studies demonstrated that Skn-1a/Pou2f3, a POU homeodomain transcription factor is expressed in these Trpm5-expressing chemosensory cells, and is necessary for their generation. Trpm5-expressing cells have recently been found in trachea, auditory tube, urethra, thymus, pancreatic duct, stomach, and large intestine. They are considered to be involved in protective responses to potential hazardous compounds as Skn-1a-dependent bitter taste cells, respiratory solitary chemosensory cells, and intestinal tuft cells are. In this study, we examined the expression and function of Skn-1a/Pou2f3 in Trpm5-expressing cells in trachea, auditory tube, urethra, thymus, pancreatic duct, stomach, and large intestine. Skn-1a/Pou2f3 is expressed in a majority of Trpm5-expressing cells in all tissues examined. In *Skn-1a/Pou2f3-*deficient mice, the expression of Trpm5 as well as marker genes for Trpm5-expressing cells were absent in all tested tissues. Immunohistochemical analyses demonstrated that two types of microvillous cells exist in trachea, urethra, and thymus, Trpm5-positive and Trpm5-negative cells. In *Skn-1a/Pou2f3-*deficient mice, a considerable proportion of Trpm5-negative and villin-positive microvillous cells remained present in these tissues. Thus, we propose that Skn-1a/Pou2f3 is the master regulator for the generation of the Trpm5-expressing microvillous cells in multiple tissues.

## Introduction

The transient receptor potential channel M5 (Trpm5) was first identified in sweet, bitter, and umami taste cells [1], and plays a critical role in taste signaling as a non-elective monovalent cation channel [2–4]. Interestingly, Trpm5-expressing cells have been also identified in several specialized cells in the extraoral tissues. For example, solitary chemosensory cells in the nasal respiratory epithelium and brush cells in the tracheal epithelium characterized by an apical tuft of microvilli exhibit taste cell-like molecular characteristics by expressing taste receptors and their downstream signaling molecules such as Gnat3, Plcb2, and Trpm5 [5–8]. These cells respond to classical bitter substances and bacterial signaling molecules (acyl-homoserine lactones), inducing protective respiratory reflexes, a neurogenic local proinflammatory response, and reduction of chemical access to the vomeronasal organ [7–12]. In the urethral epithelium, brush cells were identified to express Trpm5 as well as canonical taste receptors and taste signaling molecules. Instillation of denatonium into the urethral lumen of rats induced contraction of bladder detrusor muscle by activating Trpm5-expressing urethral brush cells to exclude noxious substrates [13]. Recent works showing that intestinal tuft cells expressing Trpm5 release interleukin-25 to initiate type II immune response and clear the gut from the parasites such as helminthes [5, 14–16] and protozoa [16] further attest that Trpm5-expressing cells function as a gatekeeper of biophylactic reactions.

Skn-1a, a POU homeodomain transcription factor also known as Pou2f3, is involved in the generation of sweet, umami, and bitter taste cells [17]. Its loss-of-function mutation expanded the sour taste cells population at the expense of sweet, umami, and bitter taste cells. Thus, Skn-1a functions as an early determinant of the differentiation to these taste cells. In addition to taste system, we previously demonstrated that Skn-1a is expressed in solitary chemosensory cells in the nasal respiratory epithelium, Trpm5-expressing microvillous cells in the main olfactory epithelium, and tuft cells in the intestine and is necessary for their generation [15,18–20]. Although Trpm5-expressing cells were also found in auditory tube [21], thymic medulla [22], pancreatic duct [23,24], and stomach and large intestine [5] functions of these cells are not well understood. Given the similarity of molecular characteristics and cellular morphology among Trpm5-expressing cells in the multiple tissues, it is presumed that Trpm5-expressing cells function as chemosensory cells in multiple tissues. Because Skn-1a is involved in the generation of all Trpm5-expressing cells examined, we hypothesize that Skn-1a is a master regulator for the generation of Trpm5-expressing chemosensory cells.

Here, we examined the expression and function of Skn-1a in Trpm5-expressing cells in multiple tissues using *Skn-1a*-deficient mice. Our results reveal that Skn-1a is expressed in the Trpm5-expressing cells in all tissues examined and is essential for generation of these cells.

## Materials and methods

### Animals

*Skn-1a*-deficient mice (*Skn-1a*-/-) were generated as described elsewhere [17]. All mice used in this study were C57BL/6 background, and 7–10 weeks old mutant and wild-type mice of either sex were used. All mouse studies were approved by the institutional animal experiment committees of the Tokyo Institute of Technology, and were performed in accordance with institutional and governmental guidelines.

### *In situ* hybridization

Probes for *Skn-1a*, *Trpm5*, *Plcb2*, *Gnat3*, *Tas1r3*, *Tas2r105*, *Tas2r108*, *Tas2r131*, and *Dclk1* were prepared as described previously [15,19,25]. Single-color *in situ* hybridization and two-color *in situ* hybridization were carried out as described previously [6,19]. Briefly, tissues were dissected from mice euthanized by CO_2_ inhalation for fresh-frozen preparation or from mice euthanized by isoflurane inhalation and transcardially perfused with 4% paraformaldehyde in PBS for fixative preparation, and were embedded in FSC22 Frozen Section Media (Leica). For single-color *in situ* hybridization, fresh-frozen tissue samples were sectioned at 8 μm thickness. Sections were fixed with 4% paraformaldehyde in PBS, treated with 0.1% diethylpyrocarbonate, prehybridized with salmon sperm DNA for 2 h at 58°C, and hybridized with antisense riboprobes for 40 h at 58°C. After hybridization, the sections were washed in 5× and 0.2× saline sodium citrate at hybridized temperature, and blocked in blocking solution containing 1.0% blocking reagent (Roche Diagnostics). Sections were then incubated with alkaline phosphatase-conjugated anti-digoxigenin antibody (1:500, Roche Diagnostics). After washing, signals were visualized with 4-nitrotetrazolium blue chloride / 5-bromo-4-chloro-3-indolyl-phosphate (Roche Diagnostics) at room temperature. For two-color *in situ* hybridization, paraformaldehyde-fixed frozen tissue samples were sectioned at 10–12 μm thickness. Sections were treated with proteinase K (3 μg/ml, Invitrogen) for 10 min at room temperature, postfixed with 4% paraformaldehyde, acetylated with acetic anhydride, and hybridized with antisense riboprobes for 40 h at 58°C. After hybridization, the sections were washed in 2×, 0.2×, 0.1× saline-sodium citrate at 58°C and blocked in blocking solution containing 0.5% blocking reagent (Roche Diagnostics). For fluorescent double labeling, the tyramide signal amplification dinitrophenyl system (PerkinElmer) was used [19]. The images were taken on an Olympus BX51 microscope with a DP71 digital CCD camera for bright-field images, and a Leica SPE confocal microscope for fluorescent images.

### Immunohistochemistry

Immunohistochemistry was performed according to a previously described method using cryosections of 10 μm thickness [19]. Tissues were dissected from mice anesthetized by isoflurane inhalation and transcardially perfused with 4% paraformaldehyde in PBS for fixative preparation, and were embedded in FSC22 Frozen Section Media (Leica). The following primary antibodies and dilutions were used: rabbit anti-Skn-1a antibody (1:500; #sc-330, Santa Cruz Biotechnology), goat anti-villin antibody (1:500; #sc-7672, Santa Cruz Biotechnology), rabbit anti-Trpm5 antibody (1:5000; #ACC-045, Alomone Labs), goat anti-ChAT antibody (1:100; #AP144P, Millipore). The following appropriate secondary antibodies were used: Alexa-488-conjugated donkey anti-rabbit IgG antibody (1:500; #A21206, Invitrogen), and Alexa-555-conjucated donkey anti-goat IgG antibody (1:500; #A11056, Invitrogen), biotin-conjugated goat anti-rabbit IgG antibody (1:500; #BA-1000, Vector Laboratories), biotin-conjugated donkey anti-goat IgG antibody (1:500; #605-706-125, Rockland).

Prior to immunostaining, we performed antigen-retrieval pretreatments in Target Retrieval Solution, pH 9.0 (Dako) for 20 min at 80°C. Following antigen-retrieval, sections were rinsed in phosphate buffered saline with 0.01% tween 20 (PBST) and blocked in 5% skimmed milk (Megmilk Snow Brand Co., Ltd.) for 1 h at room temperature, and incubated with primary antibodies overnight at 4°C. For fluorescent double labeling, sections were washed in PBST and incubated with Alexa Fluor conjugated secondary antibodies for 1h at room temperature. The sections were coverslipped with Fluomount-G including DAPI for nuclear staining (Southern Biotechnology). The fluorescent images were taken on a Leica SPE confocal microscope. For 3,3’-diaminobenzidine (DAB)-chromogenic immunostaining with streptavidin-horse radish peroxidase, sections incubated with primary antibody overnight at 4°C were washed in PBST and incubated with biotin-conjugated secondary antibodies for 1h at room temperature. The sections were rinsed in PBST and incubated in ABC solution (Vectastain ABC elite kit, Vector Laboratories) for 30 minutes according to the manufacture’s instruction. After washing, signals were visualized with 0.05% DAB (Dojindo) and 0.01% H_2_O_2_ in PBS for 5 min at room temperature.

### Reverse transcription PCR (RT-PCR)

RT-PCR was performed using tissues of wild-type and *Skn-1a-/-* mice. Trachea, thymus (one thymus lobe), urethra, auditory tube [21], and pancreatic duct were dissected from mice euthanized by CO_2_ inhalation and quickly frozen in liquid nitrogen. Total RNA was isolated from homogenized tissues individually using RNeasy mini kit (QIAGEN), and reverse transcribed using ThermoScript™ Reverse Transcriptase (Invitrogen) and oligo(dT)_20_ primer at 50°C for 120 min, and cDNA synthesis reaction was terminated by incubating at 85°C for 5min. Omission of reverse transcriptase during cDNA synthesis served as negative control. PCR was performed with Taq DNA polymerase (Takara) and primers: *Tas1r3*: 5’-catcccgtgcaacaggttc-3’ and 5’-ctggcactatagctgacctg-3’ (nucleotide 196–528, NCBI reference sequence NM031872), *Tas2r105*: 5’- gactggcttccttctcatcg -3’ and 5’- gcaaacaccccaagagaaaa -3’ (nucleotide 129–412, NM020501) [8], *Tas2r108*: 5’-tggatgcaaacagtctctgg-3’ and 5’-ggtgagggctgaaatcagaa-3’ (nucleotide 269–426, NM020502) [8], *Tas2r131*: 5’-gcagtatttataactggaatgctgg-3’ and 5’- aggcgctagttcttgtatggt-3’ (nucleotide 22–198, NM207030) [26], *Gnat3*: 5’-gtttgagcaaatcaactgccc-3’ and 5’-tcatgcattctgttcacctcc-3’ (nucleotide 71–843, NM_001081143) [26], *Plcb2*: 5’-aaagaagtgacagagccacag-3’ and 5’-ttctcctggaactgcttttcc-3’ (nucleotide 2755–3470, NM177568) [26], *Trpm5*: 5’-tcctgttcattgtgggagtcac-3’ and 5’-tggcgatcagaaggttcatg-3’ (nucleotide 2444–2926, NM020277) [27], *GAPDH*: 5’-accacagtccatgccatcac-3’ and 5’-atgtaggccatgaggtccac-3’ (nucleotide 520–986, NM008084) [27]. Conditions of cDNA amplification were 1 min at 98°C, followed by 25–40 cycles of 30 s at 98°C, 30 s at 58°C, 30 s at 72°C, and a final extension at 72°C for 5 min. GAPDH was used as a control for PCR.

### Quantitative analyses

To quantify the number of villin- and Trpm5-positive cells, every 20th transverse section (10 μm thickness) throughout the trachea (24 sections from individual mice) was collected. Urethra was transversely sectioned from bladder side at 10 μm thickness, and every 20th section was collected, and 10 sections from individual mice were used for quantitative analyses. Thymus, small intestine, and large intestine were sectioned at 10 μm thickness, and 8 sections from individual mice were used. For quantitative analyses, sections were stained using the DAB-chromogenic method as described above, and the number of signals was counted. For thymus, the densities of villin- and Trpm5-positive cells were quantified by dividing the number of positive cells by the area of sections measured by using NIH Image J. Experiments were conducted using three mice at P50–64 of each genotype, and the populations were calculated as means ± SEM (standard error of the mean), and Student’s *t*-test was used to determine the statistical significance.

For other tissues of stomach, pancreatic duct, and auditory tube, the total number of Trpm5-positive cells was counted. Three sections of stomach (gastric corpus) were obtained from individual animals of each genotype (n = 4), eight sections of auditory tube were obtained from individual animals (4 wild-type and 3 *Skn-1a-/-* mice), and four sections of pancreatic duct were obtained from individual animals (3 wild-type and 4 *Skn-1a-/-* animals). These sections were stained using the fluorescent labeling method as described above.

## Results

### The expression of Skn-1a in tracheal brush cells

**Fig 1:**
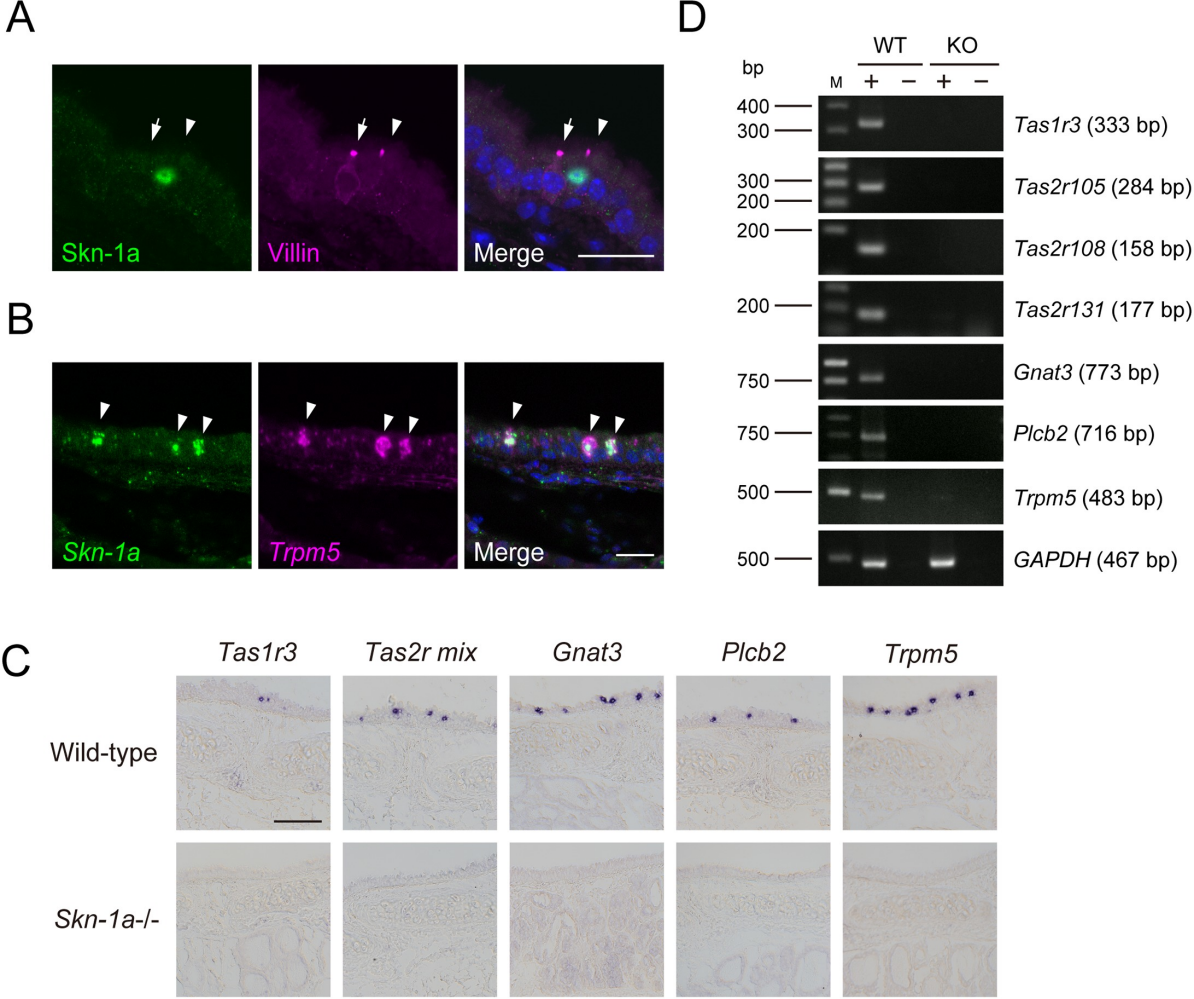
Effect of *Skn-1a* deficiency on the functional differentiation of *Trpm5*-expressing brush cells in trachea. A: Skn-1a-expressing cells were characterized using immunohistochemistry with anti-Skn-1a and anti-villin antibodies. Villin-positive brush cells were divided into two types, Skn-1a-positive (arrowhead) and Skn-1a-negative brush cells (arrow). B: *Skn-1a*-expressing cells were characterized by two-color *in situ* hybridization with RNA probes for *Skn-1a* and *Trpm5*. *Skn-1a-*positive brush cells were co-labeled with *Trpm5* riboprobe (arrowheads). Scale bars, 20 μm. C: The impact of *Skn-1a* deficiency on the functional differentiation of Trpm5/Skn1a-positive brush cells in the tracheal epithelium was examined by *in situ* hybridization using probes for taste signaling molecules of *Tas1r3*, *Tas2rs* (*Tas2r105*, *Tas2r108*, *Tas2r131*), *Gnat3*, *Plcb2* and *Trpm5*. The mRNA signals of taste signaling molecules observed in wild-type mice were completely absent in the *Skn-1a-*/- mice, indicating that *Skn-1a* is required for the functional differentiation of Trpm5-positive brush cells. Scale bar, 100 μm. D: The expression of taste signaling molecules (*Tas1r3*, *Tas2r105*, *Tas2r108*, *Tas2r131*, *Gnat3*, *Plcb2*, and *Trpm5*) in wild-type (WT) and *Skn-1a*-/- (KO) trachea was examined by RT-PCR. The expression of taste signaling molecule genes was not detected in *Skn-1a-/-* trachea. A housekeeping gene, *GAPDH* was used as a positive control.

First, we examined whether Skn-1a is expressed in brush cells in the tracheal epithelium. These cells share a common gene expression pattern with solitary chemosensory cells, including taste receptor genes and taste signaling genes (*Gnat3*, *Plcb2*, and *Trpm5*), but they are distinct from solitary chemosensory cells by apical ultrastructure and connections to nerve fibers [5–8]. To examine whether Skn-1a is expressed in tracheal brush cells, we carried out double-label immunostaining against Skn-1a combined with villin, a marker for tracheal brush cells. All Skn-1a positive cells were immunoreactive to villin, but a subset of villin positive cells was not immunoreactive to Skn-1a (Fig 1A). Because tracheal brush cells were divided into two types based on the presence or absence of choline acetyltransferase (ChAT) and ChAT-positive brush cells express *Trpm5* [8], we carried out two-color *in situ* hybridization using probes for *Skn-1a* and *Trpm5* to determine which type(s) of brush cells *Skn-1a* is expressed in. The scattered signals of *Skn-1a* mRNA were observed in the tracheal epithelium and almost all signals of *Skn-1a* mRNA were co-labeled with *Trpm5* mRNA (Fig 1B). Those results indicate that *Skn-1a* is expressed in Trpm5-positive tracheal brush cells, but not in Trpm5-negative brush cells.

### Impact of *Skn-1a* deficiency on brush cells in trachea

To investigate the function of Skn-1a in the Trpm5-expressing tracheal brush cells, we studied the impact of loss of Skn-1a function on the gene expression of the *Trpm5*-expressing tracheal brush cells that express the genes encoding taste receptors (*Tas1r3* and *Tas2rs*) and taste signaling molecules (*Gnat3*, *Plcb2* and *Trpm5*) [8,28]. *In situ* hybridization revealed that the signals for *Tas1r3*, *Tas2rs* (*Tas2r105*, *Tas2r108* and *Tas2r131*), *Gnat3*, *Plcb2* and *Trpm5* mRNA detected in wild-type mice were absent in *Skn-1a*-/- trachea (Fig 1C). RT-PCR analyses demonstrate the loss of the expression of these taste-related marker genes in *Skn-1a*-/- mice (Fig 1D). These results indicate that Skn-1a is required for the generation and/or functional differentiation of Trpm5-positive brush cells.

**Fig 2:**
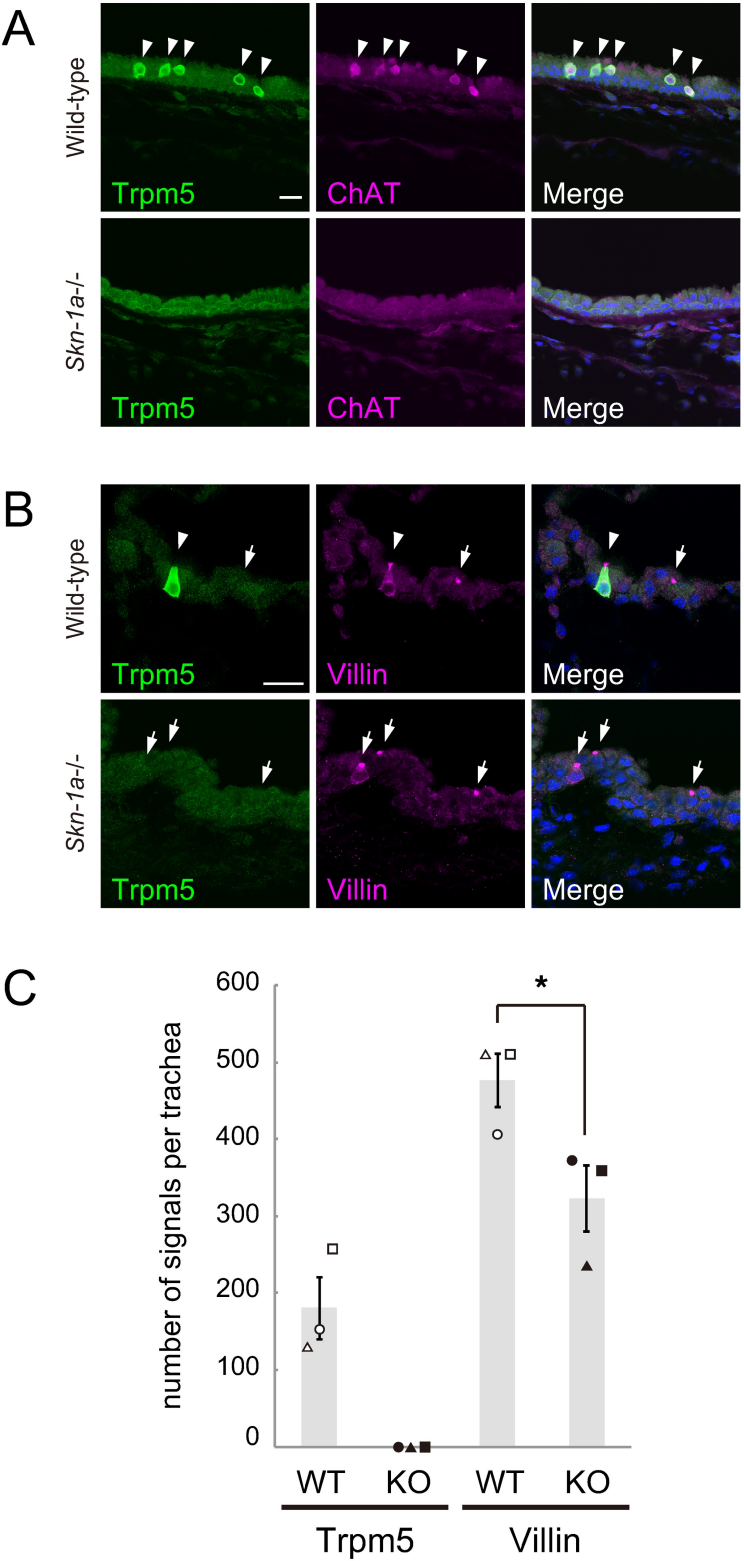
Skn-1a is required for the functional differentiation of Trpm5-positive tracheal brush cells. A: Immunostaining of Trpm5 and ChAT on coronal sections of the trachea of wild-type and *Skn-1a*-/- mice. Trpm5-positive brush cells were ChAT positive in the wild-type trachea (arrows), whereas no immunoreactive signals for Trpm5 and ChAT was observed in the *Skn-1a*-/- trachea. B: Immunostaining of Trpm5 and villin on coronal sections of the trachea of wild-type and *Skn-1a*-/- mice. In wild-type mice, both Trpm5 and villin-double positive (arrowhead) and villin-single positive (arrow) brush cells were observed. In *Skn-1a*-/- mice, Trpm5-positive brush cells were absent and only villin-single positive brush cells (arrows) were observed. Scale bars, 20 μm. C: Quantification of the number of immunosignals for Trpm5 and villin in the wild-type and *Skn-1a*-/- tracheal epithelium. The signals of Trpm5 were completely absent in the *Skn-1a*-/- tracheal epithelium, and the number of villin-single positive cells was significantly decreased in *Skn-1a*-/- mice. Each symbol represents an individual mouse. The error bars represent the mean ± SEM (n = 3, *P < 0.05, Student’s *t*-test).

To elucidate whether Skn-1a is required for the generation of Trpm5-positive brush cells, we quantified the number of Trpm5 and villin-double positive cells in the tracheal epithelium of wild-type and *Skn-1a*-/- mice by immunohistochemistry. Double label immunostaining showed no immunoreactivity to Trpm5 and ChAT in *Skn-1a*-/- mice (Fig 2A), whereas immunoreactivity to villin remained present in *Skn-1a*-/- trachea (Fig 2B). Quantitative analysis of brush cell population showed that the numbers of Trpm5- and villin-positive cells were 180 ± 41 cells and 476 ± 35 cells (mean ± SEM, n = 3), respectively, in the wild-type mice. Trpm5-positive brush cells account for about 38% of the brush cells, and approximately 62% of brush cells belong to Trpm5-negative brush cells. In *Skn-1a*-/- mice, Trpm5-positive cells were absent and the number of villin-positive cells was 323 ± 43 cells (mean ± SEM, n = 3). Compared with wild-type mice, the number of villin-positive cells in *Skn-1a*-/- mice was significantly decreased by 32% (Student’s *t*-test, p < 0.05). Because this reduction is comparable to the percentage of Trpm5-positive brush cells in all brush cells of wild-type trachea, it is conceivable that *Skn-1a* knockout diminishes the population of Trpm5-positive brush cells in the tracheal epithelium and have little effect on Trpm5-negative brush cells. These results indicate that Skn-1a functions as the critical regulator for the generation of Trpm5-positive brush cells in trachea.

### The expression and function of Skn-1a in tuft cells in digestive tracts

**Fig 3:**
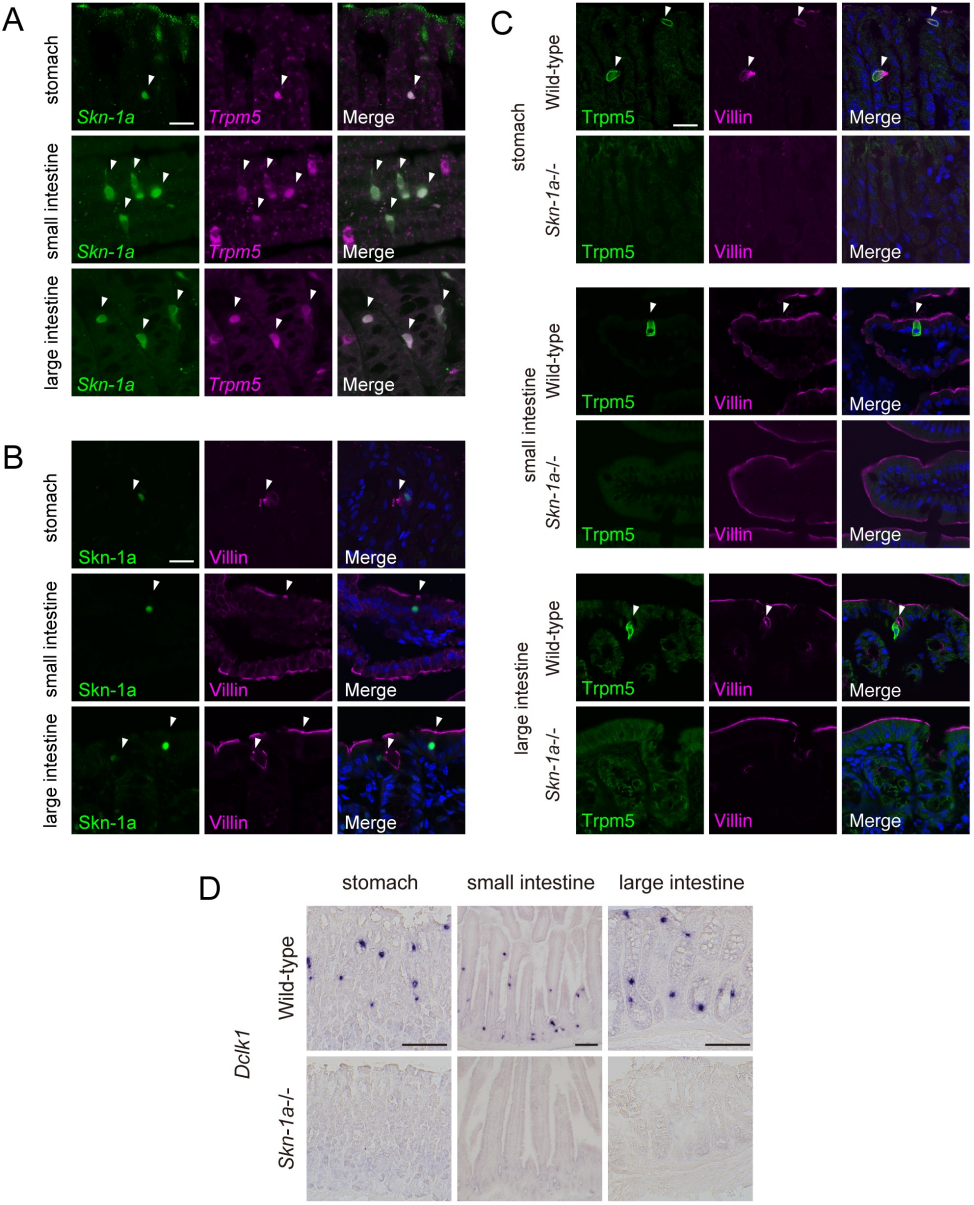
Impact of *Skn-1a* deficiency on Trpm5-positive tuft cells in digestive tracts. A: Two-color *in situ* hybridization of *Skn-1a* (green) and *Trpm5* (magenta) on sections of digestive tracts of stomach, small intestine, and large intestine of wild-type adult mice. The mRNA signals of *Skn-1a* were co-labeled with *Trpm5* signals (arrowheads) in all tissues examined. Scale bar, 20 μm. B: Co-immunostaining using antibodies against Skn-1a (green) and villin (magenta) on sections of stomach, small intestine, and large intestine of wild-type adult mice. Skn-1a-positive cells were overlapped with villin-positive cells (arrowheads). Scale bar, 20 μm. C: The impact of *Skn-1a* deficiency on Trpm5-positive tuft cells was examined by double-label immunohistochemistry of Trpm5 and villin using sections of stomach, small intestine, and large intestine of wild-type (top) and *Skn-1a*-/- mice. Trpm5-positive cells were co-labeled with anti-villin antibody (arrowheads) in wild-type mice, whereas the expression of Trpm5 was abolished in all tested tissues in *Skn-1a*-/- mice. Scale bars, 20 μm. The immunoreactive signals for villin detected in wild-type mice (arrows) were not observed in *Skn-1a*-/- mice. D: The signals of intestinal tuft cells marker gene, Dclk1 mRNA were observed in wild-type digestive tracts, whereas no signals of Dclk1 mRNA were observed in *Skn-1a*-/-. Scale bars, 100 μm.

We next examined the expression and function of Skn-1a in Trpm5-expressing cells known as tuft cells in stomach and large intestine in addition to previously reported small intestine [15, 20]. Two-color *in situ* hybridization analysis revealed that *Trpm5-*expressing tuft cells express *Skn-1a* in stomach, small intestine, and large intestine (Fig 3A). Immunohistochemical analysis showed that immunoreactivity to Skn-1a was observed in villin-positive cells (Fig 3B). These results indicate that Skn-1a is expressed in Trpm5-positive tuft cells in these digestive tracts.

To investigate the function of Skn-1a, the impact of loss of Skn-1a function on Trpm5-positive tuft cells was examined by immunohistochemistry using antibodies against Trpm5 and villin. Trpm5 and villin-double positive tuft cells observed in wild-type mice were absent in *Skn-1a*-/- mice (Fig 3C). The numbers of Trpm5 and villin-double positive tuft cells are as follows; stomach: 30 ± 4.2 cells in wild-type (mean ± SEM, n = 4), 0 in *Skn-1a-/-* (n = 4), small intestine: 563 ± 18.9 cells in wild-type (n = 3), 0 in *Skn-1a-/-* (n = 3), large intestine: 199 ± 2.72 cells in wild-type (n = 3), 0 in *Skn-1a-/-* (n = 3). To further confirm the loss of tuft cells in digestive tracts, we examined the expression of a tuft cell marker gene, *Doublecortin-like kinase 1* (*Dclk1*) [29–31]. *In situ* hybridization analysis showed that signals of *Dclk1* detected in the wild-type digestive tract were completely abolished in *Skn-1a*-/- mice (Fig 3D).

### Skn-1a acts as a master regulator for the generation of Trpm5-expressing cells

Because Skn-1a is a critical regulator for the generation of Trpm5-positive chemosensory cells in the oropharyngeal epithelium (*i*.*e*., sweet, umami, and bitter taste receptor cells), in the nasal respiratory epithelium (*i*.*e*., solitary chemosensory cells), in the main olfactory epithelium (*i*.*e*., microvillous cells), in the tracheal epithelium (*i*.*e*., brush cells), and in the stomach and intestine (*i*.*e*., tuft cells), Skn-1a may also be involved in the generation of the Trpm5-expressing cells in other tissues. To test this hypothesis, we extended our expression and functional analyses of Skn-1a to the tissues where Trpm5-expressing cells have been found, including auditory tube [21], urethra [13], thymus [22], and pancreatic duct [23,24].

**Fig 4:**
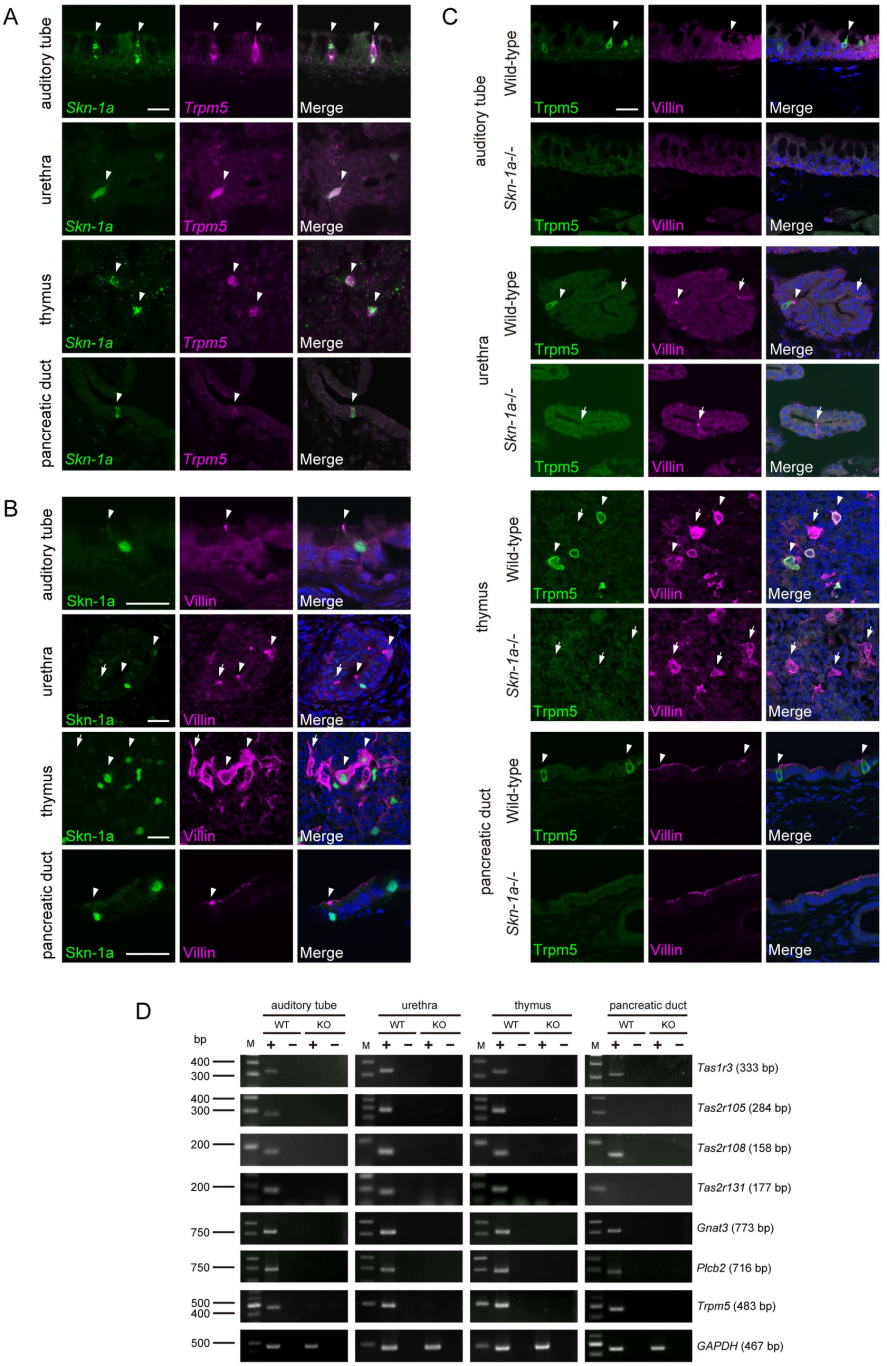
Loss of the Trpm5-positive chemosensory cells in multiple tissues in *Skn-1a*-/- mice. A: Two-color *in situ* hybridization of *Skn-1a* (green) and *Trpm5* (magenta) in various tissues of auditory tube, urethra, thymus, and pancreatic duct of wild-type adult mice. The mRNA signals of *Skn-1a* were co-labeled with *Trpm5* signals (arrowheads) in all tissues examined. Scale bar, 20 μm. B: Co-immunostaining using antibodies against Skn-1a (green) and villin (magenta) on cryosections of auditory tube, urethra, thymus, and pancreatic duct of wild-type adult mice. Skn-1a-positive cells were overlapped with villin-positive cells (arrowheads). The arrows indicate Skn-1a negative and villin-single positive cells. Scale bar, 20 μm. C: Double-label immunohistochemistry of Trpm5 and villin on sections of auditory tube, urethra, thymus, and pancreatic duct of wild-type (top) and *Skn-1a*-/- mice (bottom) was carried out to examine the impact of *Skn-1a* deficiency on Trpm5-positive chemosensory cells. Trpm5-positive cells were co-labeled with anti-villin antibody (arrowheads) in wild-type mice, whereas the expression of Trpm5 was abolished in all tested tissues in *Skn-1a*-/- mice. The immunoreactive signals for villin were detected in *Skn-1a*-/- urethral epithelium and thymic medulla (arrows), but not in auditory tube and pancreatic duct. Scale bar, 20 μm. D: The expression of taste signaling molecules (*Tas1r3*, *Tas2r105*, *Tas2r108*, *Tas2r131*, *Gnat3*, *Plcb2*, and *Trpm5*) in auditory tube, urethra, thymus, and pancreatic duct was examined by RT-PCR in wild-type (WT) and *Skn-1a*-/- (KO) mice. The expression of taste signaling molecules observed in wilt-type mice was not detected in *Skn-1a*-/- mice. A housekeeping gene, *GAPDH* was used as a positive control.

Two-color *in situ* hybridization analysis revealed that *Skn-1a* was expressed in almost all *Trpm5*-expressing cells in these tissues (Fig 4A). We also examined whether *Trpm5*-expressing cells in these tissues express villin, a marker for microvilli, that is expressed in taste receptor cells, solitary chemosensory cells, tuft cells, and brush cells. Immunosignals of Skn-1a were co-labeled with those of villin (Fig 4B). Most Skn-1a-positive cells were co-labeled with anti-villin antibody in auditory tube and pancreatic duct as well as in alimentary tract, whereas villin-single positive cells were present in urethra and thymus as well as tracheal epithelia (Fig 4B).

To further test the hypothesis, we analyzed the effects of Skn-1a deficiency on Trpm5-expressing cells by immunohistochemistry. Immunostaining using antibodies against Trpm5 and villin showed that Trpm5 and villin-double positive cells were observed in wild-type mice, whereas those were completely absent in *Skn-1a*-/- mice (Fig 4C and S1 Fig) In urethra and thymus, where Skn-1a-negative and villin-single positive cells are located, only immunosignals to villin were detected in *Skn-1a*-/- mice. The numbers of Trpm5 and villin-double positive cells are as follows; auditory tube: 9.3 ± 4.3 cells in wild-type (mean ± SEM, n = 4), 0 in *Skn-1a-/-* (n = 3), pancreatic duct: 47 ± 2.6 cells in wild-type (n = 3), 0 in *Skn-1a-/-* (n = 4), and quantitative data of urethra and thymus are summarized in S1 Fig1. These results strongly support our hypothesis that Skn-1a act as a master regulator of Trpm5-expressing microvillous cells.

In several tissues, Trpm5-expressing microvillous cells express genes encoding taste receptors and taste signaling molecules [6–9,13,16,21,22]. We examined the expression of such taste-related genes of *Tas1r3*, *Tas2r105*, *Tas2r108*, *Tas2r131*, *Gnat3*, *Plcb2*, and *Trpm5* in the auditory tube, urethra, thymus, and pancreatic duct of *Skn-1a*-/- mice by RT-PCR (Fig 4D). Taste receptor genes as well as taste signaling genes were expressed in these tissues of wild-type mice, but no taste-related genes were expressed in the *Skn-1a*-/- mice, suggesting that Trpm5-expresing microvillous cells function as chemosensory cells, and Skn-1a is required for the generation of functional Trpm5-expressing microvillous cells in these tissues.

## Discussion

In this study, we demonstrated that *Skn-1a* is expressed in Trpm5-expressing cells in all tissues examined, *i*.*e*., in trachea, urethra, auditory tube, thymus, pancreatic duct, stomach, and large intestine, in addition to sweet, umami, and bitter taste cells in the oropharyngeal epithelium [17], solitary chemosensory cells in the nasal respiratory epithelium [18], Trpm5-expressing microvillous cells in the main olfactory epithelium [19], and tuft cells in the small intestinal epithelium [15,20]. Comprehensive analyses of the loss-of-function mutation of Skn-1a across these cells revealed a crucial role of Skn-1a in generating a variety of closely related Trpm5-expressing cells in different tissues. Thus, we propose that Skn-1a functions as the master regulator for the generation of functional Trpm5-expressing chemosensory cells.

Consistent with previous observation [8], our studies showed that there are at least two types of brush cells in trachea, Trpm5 and Skn-1a double-positive brush cells and Trpm5 and Skn-1a double-negative brush cells. Trpm5 and Skn-1a double-positive brush cells have taste cell-like molecular characteristics, indicating their function as chemosensory cells. Currently, molecular characterization and function of Trpm5 and Skn-1a double-negative brush cells remain unknown. Because two types of brush cells have same cellular morphology of apical tuft of microvilli expressing villin, it is possible to speculate that they may be differentiated from a common progenitor. However, the deficiency of *Skn-1a* caused loss of Trpm5-positive brush cells and did not affect the population of Trpm5-negative brush cells, suggesting that Skn-1a is involved in the generation of Trpm5-positive brush cells but not Trpm5-negative cells, and two types of brush cells are generated from distinct cell lineages or separate from each other at earlier stage of differentiation when Skn-1a exerts its function to proceed differentiation. This is similar to the case in the main olfactory epithelium, where Skn-1a is involved in the generation of Trpm5-positive microvillous cells but not Trpm5-negative microvillous cells [19]. Trpm5-positive and Trpm5-negative microvillous cells were also observed in urethra and thymus [13, 22], and only Trpm5-negative populations remained in *Skn-1a-/-* mice. Although the numbers of villin-positive microvillous cells (Trpm5-positive and Trpm5-negative cells) in urethra and thymus were decreased in *Skn-1a-/-* mice, these reductions are not comparable to the percentages of Trpm5-positive microvillous cells in all microvillous cells of wild-type mice, suggesting the loss of Skn-1a function may affect Trpm5-nagetive microvillous cell lineages in these tissues. Identification of progenitors for Trpm5-positive brush cells and lineage-tracing experiments will provide insights into molecular mechanisms underlying Skn-1a-dependent generation of Trpm5-positive brush cells and the duality of brush cells in trachea, urethra, thymus, and main olfactory epithelium [19].

Beginning from the identification of Trpm5-expressing taste receptor cells in the oropharyngeal epithelium, function of Trpm5-expressing cells as chemosensory cells have gradually emerged in several tissues. For example, in the airway and urethral epithelium, Trpm5-expressing cells function in protective responses to potentially harmful substances (bitter substance) and bacterial products by using taste receptors [7–13]. In the intestine, Trpm5-expressing tuft cells are required for initiation of type 2 immune response to expel intruding parasites [14–16]. Although Trpm5-expressing microvillous cells in the main olfactory epithelium do not express taste-related genes except for Trpm5, these Trpm5-expressing cells are reported to function as chemo- and thermo-sensor [32], and play a protective role in maintaining the olfactory function against long-term exposure of high concentration odor mixtures [33]. The functions of Trpm5-expressing cells in auditory tube, thymus, pancreatic duct, stomach, and large intestine are currently not well understood. However, considering commonalities of molecular characteristics of Trpm5-expressing cells, it is conceivable that Trpm5-expressing cells in other tissues may also function as a gatekeeper of biophylactic reactions. Because *Skn-1a-/-* mice lack the Trpm5-expressing cells in these tissues, further studies of *Skn-1a-/-* mice would elucidate the physiological roles of Trpm5-expressing cells in mice.

## Supporting information

S1 Fig Quantitative analyses of Trpm5- and villin-positive cells in the thymus and urethra. A: Quantification of the number of Trpm5- and villin-positive cells in the wild-type and *Skn-1a*-/- urethral epithelium. Trpm5-positive cells were completely absent in the *Skn-1a*-/- urethral epithelium (8.0 ± 0.6 cells in wild-type and 0 cells in *Skn-1a*-/-), and the number of villin-positive cells tended to decrease in *Skn-1a*-/- mice compared to wild-type mice (57 ± 11 cells in wild-type and 34 ± 0.9 cells in *Skn-1a*-/-). Each symbol represents an individual mouse. The error bars represent the mean ± SEM (n = 3, P = 0.058, Student’s *t-*test). B: Densities of Trpm5- and villin-positive cells in the sections of wild-type and *Skn-1a*-/- thymus. The signals of Trpm5 were completely absent in the *Skn-1a*-/- thymus (59 ± 1.8 cells/mm^2^ in wild-type and 0 cells/mm^2^ in *Skn-1a*-/-), and the density of villin positive cells decreased significantly in *Skn-1a*-/- mice compared to wild-type mice (91 ± 3.8 cells/mm^2^ in wild-type and 64 ± 1.9 cells/mm^2^ in *Skn-1a*-/-). Each symbol represents an individual mouse. The error bars represent the mean ± SEM (n = 3, *P < 0.05, Student’s *t-*test).
(TIF)
