# Supplementary figures and images for: Skn-1a/Pou2f3 functions as a master regulator to generate Trpm5-expressing chemosensory cells in mice
Source: PLoS One. 2017 Dec 7;12(12):e0189340. doi: 10.1371/journal.pone.0189340 (PMC5720759; doi:10.1371/journal.pone.0189340)

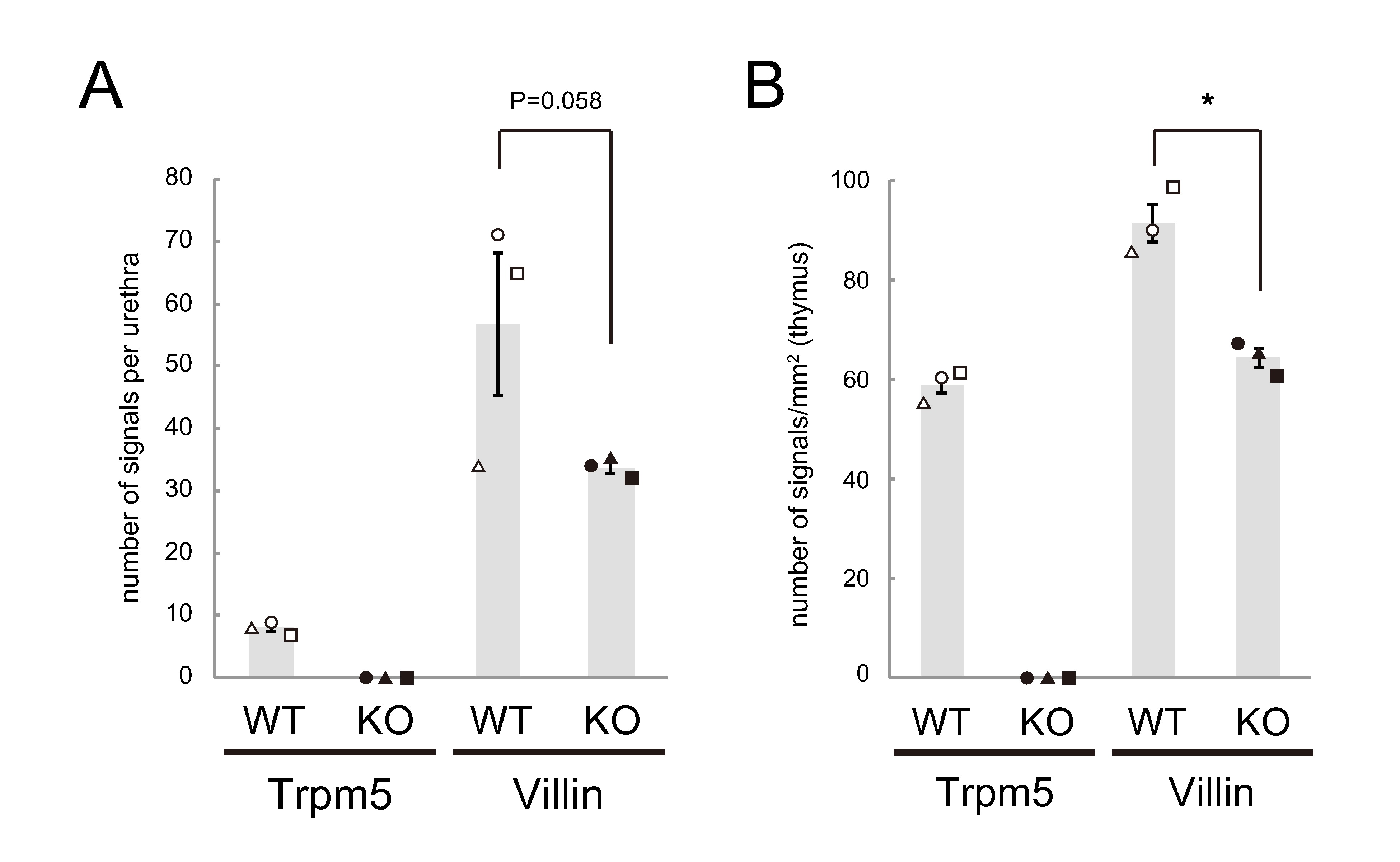

Supplement: S1 Fig — A: Quantification of the number of Trpm5- and villin-positive cells in the wild-type and Skn-1a-/- urethral epithelium. Trpm5-positive cells were completely absent in the Skn-1a-/- urethral epithelium (8.0 ± 0.6 cells in wild-type and 0 cells in Skn-1a-/-), and the number of villin-positive cells tended to decrease in Skn-1a-/- mice compared to wild-type mice (57 ± 11 cells in wild-type and 34 ± 0.9 cells in Skn-1a-/-). Each symbol represents an individual mouse. The error bars represent the mean ± SEM (n = 3, P = 0.058, Student’s t-test). B: Densities of Trpm5- and villin-positive cells in the sections of wild-type and Skn-1a-/- thymus. The signals of Trpm5 were completely absent in the Skn-1a-/- thymus (59 ± 1.8 cells/mm2 in wild-type and 0 cells/mm2 in Skn-1a-/-), and the density of villin positive cells decreased significantly in Skn-1a-/- mice compared to wild-type mice (91 ± 3.8 cells/mm2 in wild-type and 64 ± 1.9 cells/mm2 in Skn-1a-/-). Each symbol represents an individual mouse. The error bars represent the mean ± SEM (n = 3, *P < 0.05, Student’s t-test). (TIF) [file pone.0189340.s001.tif]
